# Hernia

**Published:** 1906-02-17

**Authors:** 


### PROGRESS IN MEDICINE AND SURGERY.

### HERNIA.

**Local Anæsthesia** for hernia operations is now being more extensively adopted. Corny's discovery that cocainisation of a sensory nerve trunk abolished pain-sensation throughout its area of distribution has made it possible to extend the use of local anæsthesia from minor operations into the field of general surgery. The failures which have disappointed many operators have been generally due to faulty technique. A skin incision can be painlessly made, but manipulation of deeper structures cannot be painlessly effected by simple infiltration alone. It is necessary to block the afferent nerves. The area involved in a hernia operation lends itself readily to this method. John A. Bodine,[1] from an experience of 300 operations, strongly advocates this method. He uses cocaine, the amount in no case exceeding ½ grain. A solution of 1 grain to the ounce of sterile salt solution is used. The line of skin incision is first infiltrated throughout its extent, the needle should pass through the superficial layer of the skin, and should be visible throughout; the injection must not be into the subcutaneous tissues. The needle is then passed through the anæsthetic skin and infiltration of the subcutaneous tissue made. The incision can now be painlessly made. The aponeurosis of the external oblique is now incised; it is insensitive to the cut. The ilio-inguinal nerve is found lying upon the internal oblique, and is injected as far out as possible; this is the largest and most important nerve and the easiest to find. The ilio-hypogastric and genito-crural are injected if found; if they cannot be found further injections of the tissues will be necessary. These two are, however, of minor importance; if they can be found, and are injected, the operation can be rendered quite painless. This method is of very great value in strangulated hernia with intestinal obstruction and fæcal vomiting, which is a most dangerous condition for general anæsthesia, deaths from inspired vomit in such cases are probably within the experience of all. A solution of eucaine and adrenalin such as recommended by A. E. Barker [2] is probably more popular than cocaine, and appears to be equally effectual; it gives rise to no fears of toxic symptoms, although Bodine says the amount of cocaine he uses never gives rise to such symptoms. An injection of morphia is useful, and should cocaine have been used, morphia acts as an antidote were poisoning possible. For the cure of femoral hernia many methods have been devised; the experience of most surgeons being that recurrence is more liable to follow operation in this form than in inguinal hernia. It is of interest therefore to hear that by a very simple method a good result can be assured. W. Burton de Garmo [3] has treated 110 consecutive cases by a single method, and has had only one recurrence. Three of his cases had been operated on previously by other surgeons, and have now been free from recurrence for more than four years. After ligature of the sac, Poupart's ligament is sewn down by means of kangaroo tendon passed with a blunt needle, though all structures down to the periosteum of the pubes. The ring is thus closed. The patients are allowed up on the tenth day. There is nothing new in the method, but the fact that so many cases have been effectually cured by so simple a procedure is of interest.

**Torsion of the Great Omentum.**—Edred M. Corner and Pinches [4] contribute an article on this comparatively rare and but recently recognised condition. Torsion is frequently recognised in the loop of bowel in a strangulated hernia; when the bowel is reduced the contained gas is sufficient to untwist it. Torsion of the omentum takes place in the same way. The total number of cases so far recorded is 53—mostly since the year 1900. The cases may be classified clinically into three groups: (1) abdominal: There is here no hernia present; six examples recorded. (2) Hernial: Torsion within the sac; six examples. (3) Hernial and abdominal: Torsion not limited to the sac, but extends into the abdomen; 41 cases recorded. Of the first group five cases were diagnosed as appendicitis, with probably local abscess. Of the second group four were diagnosed as strangulated hernia. The other two as irreducible and incarcerated herniæ respectively. Of the third group 14 were thought to be strangulated herniæ, 9 appendicitis, 2 reduction "en masse," and among other diagnoses were sarcoma of testis, abdominal tumour, chronic peritonitis, strangulated umbilical hernia, intra-peritoneal abscess, and one was correctly diagnosed as torsion of omentum. The presence of a strangulated inguinal hernia in a female should raise the suspicion of the presence of a uterine appendage or twisted omentum in the sac. In 90 per cent. of cases an inguinal hernia was present; in no case was there a femoral hernia. The hernia is commonly one of long standing. Many twists, sometimes ten, have been found. In many cases there was a history of previous attacks of pain, the twists appeared to have been formed by slow increments, and the previous attacks of pain may probably be referred to the occurrence of these repeated torsions. As to clinical symptoms, the onset in 37.7 per cent. was sudden, in 13.2 per cent. gradual, in others not stated. Pain is the most constant symptom, and the first to appear. Vomiting does not appear to be very constant; it is said to have been present in 35.8 per cent., in 26.4 per cent. its absence is noted, in 37.7 per cent. it is not mentioned. The condition of the bowels is variable; it is generally one of inactivity. The pulse is accelerated, varying from 80 to 120. The temperature is slightly raised.

**Clinical Signs.**—A tumour was present in all but three, its situation varied. In (1) abdominal group: Mass in right iliac fossa. (2) Hernial group: Hard painful irreducible swelling in inguinal region. (3) Hernial and abdominal: Hernial tumour only 12 cases; abdominal tumour, 7 cases; tumour in both situations, 19 cases; no tumour, 3 cases.

**Prognosis.**—All but eight recovered. Of these 3 died of pneumonia, 1 of peritonitis after operation; two were too bad to attempt operation. The condition is one not yet sufficiently widely recognised to enable us to say much about diagnosis. The writers thus summarise the conditions which should lead to a suspicion of torsion of the omentum. The subject is generally an elderly man, with an old standing inguinal hernia, which has become troublesome and gives rise to certain symptoms which can be summed up as those of subacute intestinal obstruction. Vomiting may be absent and both fæces and flatus passed. The hernia will appear painful and irreducible, incarcerated or strangulated. Usually a tumour is to be felt both in the scrotum and the abdomen. Occasionally only the abdominal tumour is present, the sac appearing to be empty. For the relief of the condition it may be necessary to open the abdomen as well as the hernial sac. In a certain number of cases the hernia was reduced without relief of symptoms, although the sac was empty. In a few instances the attack came on when the sac was empty, and in these cases there is no definite guide to correct diagnosis.

[1] Med. Rec., Oct. 21, 1905. [2] Brit. Med. Jour., Dec. 24, 1906. [3] Annals of Surg., Aug., 1905. [4] Amer. Jour. Med. Sc., Aug., 1905.

